# Artificial Intelligence based Models for Screening of Hematologic Malignancies using Cell Population Data

**DOI:** 10.1038/s41598-020-61247-0

**Published:** 2020-03-16

**Authors:** Shabbir Syed-Abdul, Rianda-Putra Firdani, Hee-Jung Chung, Mohy Uddin, Mina Hur, Jae Hyeon Park, Hyung Woo Kim, Anton Gradišek, Erik Dovgan

**Affiliations:** 1 Graduate Institute of Biomedical Informatics, Taipei Medical University, Taipei, Taiwan; 2 Department of Laboratory Medicine, Konkuk University School of Medicine, Seoul, South Korea; 3 Executive Office, King Abdullah International Medical Research Center, King Saud bin Abdulaziz University for Health Sciences, Ministry of National Guard - Health Affairs, Riyadh, Kingdom of Saudi Arabia; 4 Department of Laboratory Medicine, Seoul National University Hospital, Seoul, South Korea; 5 Department of Internal Medicine, College of Medicine, Institute of Kidney Disease Research, Yonsei University, Seoul, South Korea; 6 Department of Intelligent Systems, Jožef Stefan Institute, Jamova cesta 39, SI-1000 Ljubljana, Slovenia; 7 CPBMI Consortium, Biomedical Informatics Training and Education Center of Seoul National University Hospital, Seoul, South Korea

**Keywords:** Cancer screening, Haematological diseases, Haematological cancer, Information technology

## Abstract

Cell Population Data (CPD) provides various blood cell parameters that can be used for differential diagnosis. Data analytics using Machine Learning (ML) have been playing a pivotal role in revolutionizing medical diagnostics. This research presents a novel approach of using ML algorithms for screening hematologic malignancies using CPD. The data collection was done at Konkuk University Medical Center, Seoul. A total of (882 cases: 457 hematologic malignancy and 425 hematologic non-malignancy) were used for analysis. In our study, seven machine learning models, i.e., SGD, SVM, RF, DT, Linear model, Logistic regression, and ANN, were used. In order to measure the performance of our ML models, stratified 10-fold cross validation was performed, and metrics, such as accuracy, precision, recall, and AUC were used. We observed outstanding performance by the ANN model as compared to other ML models. The diagnostic ability of ANN achieved the highest accuracy, precision, recall, and AUC ± Standard Deviation as follows: 82.8%, 82.8%, 84.9%, and 93.5% ± 2.6 respectively. ANN algorithm based on CPD appeared to be an efficient aid for clinical laboratory screening of hematologic malignancies. Our results encourage further work of applying ML to wider field of clinical practice.

## Introduction

The global burden of blood cancers is rising and it has affected the lives of millions of people with all ages globally. Hematological malignancies have a major contribution in disease burden almost in every country. The status report produced by the International Agency for Research on Cancer (IARC) estimated 18.1 million new cancer cases and 9.6 million cancer deaths in 2018; 1 out of 5 men and 1 out of 6 women get cancer in their life, and 1 out of 8 men and 1 out of 11 women die due to cancer; and the estimated 5 year prevalence of cancer is 43.8 million^1^. According to the detailed systematic analyses from Global Burden of Disease Cancer Collaboration, the current cancer trends pose a threat to human development, and if these trends continue then the cancer incidence and prevalence are expected to increase in the future due to population growth, ageing and epidemiological transitions^2,3^. These facts highlight the importance and urgency of implementing efficient prevention and early detection policies for cancer along with the strategic investments and effective programs for cancer control in order to provide universal access to cancer care and achieve the global health action plans^2,4^. Clinical and biological classifications have been developed by the World Health Organization (WHO) to recognize, categorize and treat the hematological malignancies^5^. Various clinical methods and techniques, such as biopsies, blood tests, immunology tests, flow cytometry, radiology exams, as well as genetics technologies, such as chromosome analysis and DNA sequencing exist for the diagnosis of hematological malignancies^6,7^. Complete Blood Count (CBC) is one of the basic and fundamental tests to evaluate a variety of health disorders including hematological malignancies. With technological innovations, the Next-Generation Hematological Analyzers (HA) are instrumental in cellular and morphological analysis^8^. Though these analyzers are most commonly used for cell counts and differential leukocyte analysis, but their maximum potentials still need to be utilized^8^. They can provide additional parameters to support the screening and diagnosis of different diseases, e.g. they can expand the potential information from CBC^8,9^. The Cell Population Data (CPD) generated from these analyzers provides various blood cell parameters and have proved its usefulness in the screening of hematological and non-hematological diseases^10^. The literature has provided successful examples of utilizing clinical information using CPD parameters for diagnosis and management of infectious diseases, such as Sepsis^9,10^.

With the advent of time, latest Information and Communication Technologies (ICTs) are paving the way for new discoveries of screening, diagnosing and predicting diseases; and Artificial Intelligence (AI) is one of the most influential names in that technological list. AI is the field of computer science that simulates human intelligence by creating intelligent machines. It has great potentials to identify the relevant clinical information that is hidden in large scale or big healthcare data. AI and its branches, such as Machine Learning (ML) have made remarkable achievements in healthcare industry in the past decades and have been playing a pivotal role in revolutionizing the medical diagnostics and practices through intelligent applications and tools. Some important uses of ML applications in clinical practice include: provision of up-to-date information for reducing diagnostic and therapeutic errors, real time inferences, health risk alerts, and health outcome predictions^11,12^. Though there is substantial literature of AI and ML in healthcare research, most of the research focuses in the fields of Cancer, Neurology and Cardiology^11,13–21^. In addition, the literature lacks successful applications of ML that deal with complex medical diagnostic fields like Hematology^22^. Blood tests are the most common measure to diagnose the hematological diseases in the laboratories and clinicians need the hematological parameters to analyze the numerical patterns, deviations and relations; and that’s where ML algorithms can come into action by performing intelligent handling, detection and utilization of these parameters, and developing models to predict the future diagnosis and outcomes^22^.

This research presents a novel approach of using ML algorithms for screening patients for hematologic malignancies using CPD. The term screening refers to the medical process of determining the likelihood of disease in healthy population; and based on subsequent diagnostic tests or procedures, it can lead to the intervention / treatment of the diagnosed disease. Therefore, our proposed approach is not and cannot be used for diagnosing or treating the malignancies, rather it just provides a simple technological support for screening the patients using their numerical data. In order to measure the performance of ML models, stratified 10-fold cross validation was performed, and metrics like accuracy, precision, recall, Area Under the Curve (AUC), and Receiver Operating Characteristic (ROC) were used.

## Methods

This study was performed in Konkuk University Medical Center (KUMC), which is 700-bed sized tertiary-care teaching hospital in Seoul, South Korea. The study was conducted according to the Declaration of Helsinki, the protocol approved an exemption by the Institutional Review Board (IRB) of KUMC, and obtaining informed consent from the study patients was not necessary (IRB approval No. KUH1200110). The data collection was done at the Department of Laboratory Medicine, Konkuk University Medical Center from February 2019 to March 2019. The data was anonymized due to the sensitivity of patients’ information. CPD parameters and International Classification of Diseases, 10th Revision (ICD-10) codes were included. The demographic patient information, i.e., gender and age, were also included for better prediction outcomes.

We performed the hematologic analysis using Mindray BC-6800 (Mindray, Shenzhen, China) automated hematology analyzer that yielded CPD including CBC, leukocyte differentiation and reticulocyte count with information on volume, conductivity and different scatter measures^23^. After preprocessing (see the following section), a total of 882 cases were included for analysis. Detailed number of hematologic diseases including malignancies and non-malignancies are shown below in Table 1.

**Table 1:** Case numbers analyzed in the study (after preprocessing).

ICD-10 Code	Type	Cases	Group
C81-C96	Malignant neoplasms of lymphoid, haematopoietic and related tissue	457	Hematologic - Malignancies
D50-D53	Nutritional anemia	49	Hematologic – Non Malignancies
D55-D59	Haemolytic anemia	6	Hematologic – Non Malignancies
D60-D64	Aplastic and other anemia	166	Hematologic – Non Malignancies
D65-D69	Coagulation defects, purpura and other hemorrhagic conditions	83	Hematologic – Non Malignancies
D70-D77	Other diseases of blood and blood-forming organs	121	Hematologic – Non Malignancies

### Preprocessing

The dataset contained several missing values. We handled this issue in two steps. First, the cases that had more than 90% values missing were excluded. In total, 17 cases were excluded, while 882 cases were further analyzed. Second, missing values were predicted with two machine learning algorithms. The missing numerical variables were predicted with linear regression, while the missing categorical variables were predicted with decision tree classifier. In both cases, the learning data contained a subset of numerical attributes and a subset of instances with no missing values.

After handling missing values, we selected only laboratory data and demographic patient information (gender and age) for further analysis. As a result, the number of variables (before feature selection) was 61.

There are different ranges of measurements and units in the laboratory data, therefore, in order to normalize our dataset, we used the scaling process. We selected Min-Max Scalar as scaling feature to transform the normal values to end up within the range of 0 to 1. In order to make the gender values in numerical form, we used the value of 0 for female and 1 for male.

### Bias variable

We applied point-biserial correlation to determine which variables have significant influence on malignant or non-malignant hematologic diseases. Point-biserial correlation is assessed between −1 to 1. The value closer to −1 shows the strong confidence of negative linear relationship between two variables, and the value closer to 1 shows strong confidence of positive linear relationship.

The presented approach uses filter-based variable/feature selection. However, there exist two additional approaches for selecting the most appropriate features: wrapper and embedded approaches. The main differences among them are the following. Filter methods use a selected measure to get the best subset of features prior machine learning phase. Wrapper methods use machine learning model to score the feature subsets and select the best performing one. Embedded methods perform feature selection as a part of model construction process.

### Variable selection

In order to find out the variables with high significance, either negative or positive point-biserial correlation, we used the absolute value by changing the results from negative correlation to positive value, and ranked them from high to low. Table 2 shows the selected variables based on point-biserial correlation.

**Table 2:** CPD selected variables based on point-biserial correlation.

Abbreviation	Name	Absolute Correlation
P-LCC	Platelet-large cell count	0.351
PCT	Plateletcrit	0.336
PLT	optical impedance	0.321
PLT-I	Platelet count- Impedance	0.320
InR‰	Infected RBC percentage	0.297
Age	Age	0.282
Gender	Gender	0.231
HFC%	High fluorescent Cell percentage	0.223
Neu-BF%	Neutrophils percentage -body fluid	0.210
H-NR%	High forward scatter NRBC ratio	0.198
PLR	Platelet-to-lymphocyte ratio	0.188
Neu-BF#	Neutrophils Number -body fluid	0.186
HF-BF#	High Fluorescent cell Number -body fluid	0.181
NLR	Neutrophil-to-lymphocyte ratio	0.181
L-NR%	Low forward scatter NRBC ratio	0.179
Mon%	Monocytes percentage	0.168
MO-BF%	Monocytes percentage- body fluid	0.166
LY-BF%	Lymphocytes percentage- body fluid	0.157
Eos-BF#	Eosinophils number -body fluid	0.152
RDW-CV	Red Blood Cell Distribution Width Coefficient of Variation	0.149
IMG%	Immature Granulocyte percentage	0.146
Micro#	RBC microcyte Cell Number	0.143
Micro%	RBC microcyte Cell percentage	0.142
RDW-SD	Red Blood Cell Distribution Width Standard Deviation	0.141
Macro#	RBC macrocyte Cell Number	0.130
HCT	Hematocrit	0.128
IME%	Immature eosinophil percentage	0.114
HGB	Hemoglobin Concentration	0.110
MCHC	Mean Corpuscular Hemoglobin Concentration	0.100
RBC	Red Blood Cell count	0.098
Macro%	RBC macrocyte Cell percentage	0.096
Lym#	Lymphocytes number	0.095
MPV	Mean Platelet Volume	0.093
MCV	Mean Corpuscular volume	0.091
LY-BF#	Lymphocytes number- body fluid	0.090
Bas%	Basophils percentage	0.089
MO-BF#	Monocytes number- body fluid	0.084
P-LCR	Platelet-large cell ratio	0.075
Eos-BF%	Eosinophils percentage -body fluid	0.064
NRBC#	Nucleated red blood cell number	0.059
NRBC%	Nucleated red blood cell percentage	0.057

### Model selection

In our study, we applied seven machine learning models: Stochastic Gradient Descent (SGD), Support Vector Machine (SVM), Random Forests (RF), Decision Tree (DT), an adapted Linear Regression – its output was discretized into two classes by using a threshold – (LINEAR), Logistic Regression (LOGIT), and Artificial Neural Networks (ANN). The first six models were used from the Scikit-learn library^24^ with the default parameter values, while ANN used the Keras library^25^.

ANN consisted of a 3-layer architecture and was trained in 300 epochs with batch size 48. The first hidden layer had 128 nodes with Rectified Linear Unit (ReLU) activation function, and the second hidden layer had 64 nodes with ReLU activation function. A single node with Sigmoid/Logistic activation was used for the output layer. The output layer was defined as malignancies predictive value, which is a continuous variable from 0 (haematologic non-malignancies) to 1 (haematologic malignancies). This architecture was selected based on our past experience on processing similar medical datasets. A more appropriate approach for the selection of the architecture would include evaluation of various parameter values (such as number of layers). However, such an optimization is very complex and time-consuming thus will be carried out in future work if deemed necessary.

### Performance evaluation

To evaluate the performance of the ML models, we used the stratified 10-fold cross-validation. In stratified cross-validation, the folds are selected in such a way that the percentage of samples is preserved for each class^26^. That is, the procedure maintains the same distribution of the target variable when randomly selecting examples for each fold; in our case, the same proportion between malignant and non-malignant cases. More precisely, this procedure divides the set of cases into k groups (k = 10) or folds of approximately equal sizes. The first fold is treated as a testing set, and the remaining k-1 folds are used for training the model (90% training data vs. 10% testing data). This is repeated 10 times, each time selecting a different fold as the testing set and the remaining folds as the training set. The performance metrics are then averaged over all the 10 steps. To avoid double dipping, training and testing sets (folds) are always disjoint sets and thus they do not share any sample^27^.

In our study we tested data with True Positive (TP) as real malignancies that are correctly predicted, False Positive (FP) as real malignancies that are incorrectly classified to be non-malignancies, True Negative (TN) as real non-malignancies that are correctly predicted, and False Negative (FN) as real non-malignancies that are incorrectly predicted. The results of tested performance measures from precision denotes the proportion of predicted positive cases or TP. Recall refers to sensitivity, and in medical term to identify all positive cases or rate of TP. Accuracy is predicting the correct ratio of samples, and is one of the most intuitive and basic performance measures for any ML model. Area Under the Curve (AUC) is used to determine the best cutoff point and compare two or more tests or observers of each calculated fold^28^. AUC compares rate of TP (TPR) and rate of FP (FPR). It is created by plotting the TPR against the FPR^29^.

## Results

Comparative analysis of gender on malignant and non-malignant group revealed different results. We found that males in our set have a higher ratio in malignancies with 277 cases, as opposed to females with 180 cases. Among non-malignant groups that had opposite results, females had higher ratio with 266 cases than males with 159 cases. The demographic population distribution on malignant and non-malignant group is shown in Table 3.

**Table 3:** Demographic population distribution.

Age	Malignancies		Non-Malignancies		Total (%)
	Female (%)	Male (%)	Female (%)	Male (%)	
<18 (Children)	0 (0)	3 (0.34)	1 (0.11)	1 (0.11)	5 (0.57)
18–64 (Adults)	124 (14.06)	207 (23.47)	152 (17.23)	63 (7.14)	546 (61.90)
65 + (Elderly)	56 (6.35)	67 (7.60)	113 (12.81)	95 (10.77)	331 (37.53)
Total	180 (20.41)	277 (31.41)	266 (30.16)	159 (18.03)	882 (100)

The classification information from our dataset was placed into two groups, haematologic malignancies and haematologic non-malignancies using ICD-10 code. As shown in Table 4, C92 or myeloid leukemia disease had the highest percentage (20.07) in malignant group with 177 cases, in which 167 cases belonged to acute myeloid leukemia disease. In Non-Malignant group, D64 Pancytopenia took the highest cases with a total of 106 followed by D61 with 60 cases.

**Table 4:** Granularity information of group diseases in dataset.

Group	ICD code	Disease category	Frequency	Percentage (%)
**Malignant Group**	C81	Hodgkin lymphoma	9	1.02%
	C82	Follicular lymphoma	1	0.11%
	C83	Non-follicular lymphoma	50	5.67%
	C84	Mature T/NK-cell lymphomas	10	1.13%
	C85	Other specified and unspecified types of non-Hodgkin lymphoma	28	3.17%
	C86	Other specified types of T/NK-cell lymphoma	22	2.49%
	C88	Malignant immunoproliferative diseases and certain other B-cell lymphomas	15	1.70%
	C90	Multiple myeloma and malignant plasma cell neoplasms	20	2.27%
	C91	Lymphoid leukemia	73	8.28%
	C92	Myeloid leukemia	177	20.07%
	C94	Other leukemias of specified cell type	13	1.47%
	C95	Leukemia of unspecified cell type	38	4.31%
	C96	Other and unspecified malignant neoplasms of lymphoid, hematopoietic and related tissue	1	0.11%
**Non-Malignant Group**	D50-D53	Nutritional anaemias	49	5.56%
	D55-D59	Haemolytic anaemias	6	0.68%
	D60-D64	Aplastic and other anaemias	166	18.82%
	D65-D69	Coagulation defects, purpura and other haemorrhagic conditions	83	9.41%
	D70-D77	Other diseases of blood and blood-forming organs	121	13.72%
**Malignant Group**	C81-C96		457	51.81%
**Non-Malignant Group**	D50-D77		425	48.19%
**Total**			882	100%

The performance of the ML models was measured with 10-fold cross-validation as described in Section Performance Evaluation. In addition to the ML models, we also evaluated variable selection with thresholds 0.05, 0.1, 0.15, and 0.2 (see Table 5). When evaluating a threshold, all the variables with lower absolute point-biserial correlation were removed from the dataset. The results show that, for all the tested thresholds, the highest AUC is obtained by ANN. In addition, since there is low difference in AUC when applying the threshold of 0.05 in comparison to when no threshold is applied, the recall was also evaluated and the results show that recall is the highest when the threshold of 0.05 is applied. Consequently, we selected variable selection with the threshold of 0.05 for further analysis. Such a variable selection eliminated 20 variables, as shown in Table 5.

**Table 5:** Total variable predictor on selection variable and model with high result AUC and recall.

Used variable	Total Variable Predictor	Model	AUC % (± Standard Deviation)	Recall
All Variables	61	ANN	93.9 ± 3	84.2
>0.05	41	ANN	93.5 ± 3	84.9
>0.1	29	ANN	92.8 ± 3	83.6
>0.15	19	ANN	90.7 ± 5	82.8
>0.20	9	ANN	87.7 ± 5	79.1

**Figure 1:**
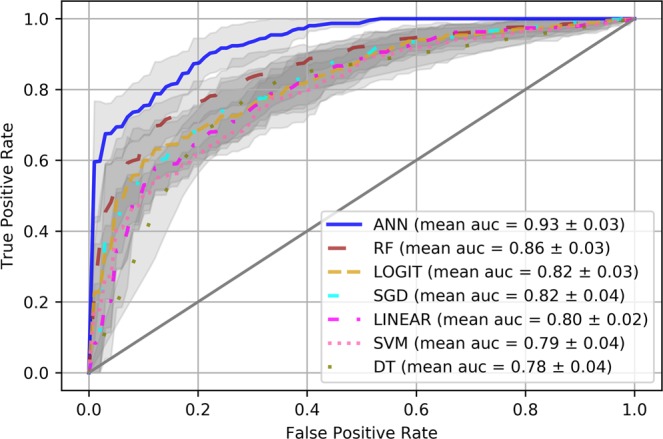
AUC Obtained with ML Models when Applying Variable Selection with the Threshold of 0.05.

The results of all the ML models when applying variable selection with the threshold of 0.05 are shown in Fig. 1 and Table 6. These figure and table show that ANN has the best performance among ML algorithms. More precisely, the diagnostic ability of ANN achieved the highest accuracy, precision, recall (diagnostic sensitivity) and AUC ± Standard Deviation as follows: 82.8%, 82.8%, 84.9%, and 93.5% ± 2.6 respectively.

**Table 6:** Model performance indicators when applying variable selection with the threshold of 0.05.

	AUC ± Standard Deviation	Accuracy	Precision	Recall
Stochastic Gradient Descent (SGD)	0.823 ± 0.040	0.699	0.746	0.710
Support Vector Machine (SVM)	0.792 ± 0.035	0.716	0.719	0.744
Decision Tree (DT)	0.782 ± 0.039	0.728	0.745	0.722
Ramdom Forest (RF)	0.859 ± 0.027	0.778	0.803	0.764
Linear Regression (LINEAR), adapted	0.802 ± 0.019	0.721	0.726	0.742
Logistic Regression (LOGIT)	0.822 ± 0.034	0.725	0.741	0.724
Artificial Neural Network (ANN)	0.935 ± 0.026	0.828	0.828	0.849

For the statistical comparison of the algorithms, we applied Dietterich’s 5×2-Fold Cross-Validation method^30^. This method performs K-fold paired *t* test in order to compare the performance of two algorithms. The statistical comparison of the algorithms is shown in Table 7. This table shows that, assuming the level of significance of 0.05, the performance of ANN is significantly different with respect to the performance of other models.

**Table 7:** The *p* values of testing hypothesis that pairs of algorithms perform similarly.

	SVM	DT	RF	LINEAR	LOGIT	ANN
**SGD**	0.329	0.497	0.238	0.577	0.773	0.019
**SVM**		0.187	0.051	0.123	0.165	0.010
**DT**			0.161	0.304	0.892	0.002
**RF**				0.099	0.104	0.000
**LINEAR**					0.507	0.010
**LOGIT**						0.005

## Discussion

An ample amount of research has been done by utilizing AI and patients’ clinical information for diagnosis and management of various diseases. Here, our comparative analysis will focus on AI based studies in the literature that have utilized CBC and particularly CPD for screening hematologic malignancies. The morphological identification of blood cell disorders with CPD is critical for the early diagnosis and clinical decision. Accordingly, CPD could be used to assist physicians who are not specialized in haematology by facilitating the CBC and suggesting proper and early patient referral. In a study using CBC test data, three data mining methods: association rules, rule induction and deep learning were tested and the results showed that the deep learning classifier with the best ability for predicting tumors from blood diseases with an accuracy of 79.45%, with the limitation of no explanation of results^31^. Another related study^32^ used machine learning algorithm to differentiate lymphoid classification using CPD parameters from 3 cohorts: healthy control, viral infection and chronic lymphocytic leukemia. In that study, the best result came from Neural Networks classifier with an accuracy of 98.7% followed by SVM 98.0% and KNN 98.0%^32^. A recent study using CPD showed Random Forest algorithm as the best model with two practices, using all parameters and reduced parameters. It showed the accuracy of 59% for 181 parameters and accuracy of 57% for 61 parameters^22^. Another study took CPD data with 103 parameters for prediction of relapse in childhood with Acute Lymphoblastic Leukemia^33^. It showed the Random Forest as the best model for prediction with measurements (accuracy: 83.1%; specificity 89.5%; positive predictive value: 88.0% and AUC: 90.2%). One slightly different retrospective study^34^ in the field of medical imaging with 467 cases (training set: 360 and test set: 107) constructed SVM texture classifier model to see the feasibility of differentiating bone marrow with hematologic diseases. With the above-mentioned training set, the values of accuracy, sensitivity and specificity and AUC were 82.8%, 81.7%, 83.9% and 0.895 (*p* < 0.001) respectively. The model’s predictive performance was comparable to the radiologists, but it requires more clinical and lab work for the finalization.

In our study, the results showed that machine learning approach, using deep learning algorithm trained on large amount of multi-analyte sets from laboratory blood test results, is able to predict diseases with high accuracy. Under these conditions, a classification (diagnostic) accuracy of 82.8% (ANN) and AUC 93.5% for the two classifications represent excellent results; and ANN are comparable to that of other ML methods have significance improvement.

Moreover, our results showed that the step of filtering variables based on point-biserial correlation had better results. Total variable predictor without filtered by point-biserial correlation would contain weak association with the classes. This suggests that there is a bias to assess malignancies and non-malignancies classes for choosing variables on CPD. Therefore, the highest selection that eliminated predictor with point-biserial correlation below 2 is not covered as outstanding result because AUC performance decreased from 93.9 to 87.7% (see Table 5). This type of predictor has an excellent selection but it has to be filtered to eliminate the weak association of the variable. Our results encourage further work of applying machine learning to the wider field of internal medicine.

As far as the association of platelet-large cell count with malignancy is concerned, a high blood platelet count is a strong predictor of cancer and should be urgently investigated further. A high platelet count may be referred to as thrombocytosis. This is usually the result of an existing condition (also called secondary or reactive thrombocytosis), such as: cancer - most commonly lung, gastrointestinal, ovarian, breast or lymphoma. Also, optimal impedance (PLT) is an advanced technique that provides an accurate automated complete blood count (CBC), including white blood cell (WBC) differential, in a short turnaround time. Clinically, it makes sense that PLT is among top influential variables in the model.

There were certain limitations in our study. It used a relatively small sample size and many cases were excluded for the main purpose of the study. The data collection process took two months, and we excluded data due to the machine learning algorithm restriction of high missing items. Due to the sample size, we focused on CPD and used the stratified cross-validation method. Moreover, we did not perform validation with external data, as we worked with the accumulated dataset only. Hence, the diagnostic ability of machine learning using other external data, e.g. (gene expression data) should be applied in the future. Our study mainly focused on predictive accuracy and did not look at the other additional benefits from CPD. For future investigations, we suggest the following potential areas for further investigation: predictive performance, counting identifying tasks and metrics, testing different approaches for data modeling, and understanding portions of the data have contrasting contributions to predictive accuracy. Furthermore, since genomic analysis is already a part of the clinical practice for the diagnosis and management of diverse hematologic malignancies, so the genomic evaluation of cancer supported by upcoming improvements in molecular diagnostic technologies is another key area that must be considered for the future research.

## Conclusions

This research presents a novel approach of using ML algorithm for screening patients with suspected hematologic malignancies versus non-malignancies using CPD that was generated by routine CBC. We observed outstanding performance results on ANN model, as the diagnostic ability of ANN achieved higher accuracy, prediction, recall and AUC as compared to the other ML models. Therefore, we conclude that based on CPD, the ANN algorithm appears to be an efficient aid for the clinical laboratory diagnostic approach of hematologic malignancies. In the future, we are planning to apply this algorithm to the outpatient data in hematology departments. Prospective research and trials are mandatory to confirm the validity of clinical AI before it actually helps physicians in clinical practice, particularly in haematologic diseases.
