# Author Correction: Artificial Intelligence based Models for Screening of Hematologic Malignancies using Cell Population Data

**DOI:** 10.1038/s41598-020-67559-5

**Published:** 2020-06-23

**Authors:** Shabbir Syed-Abdul, Rianda-Putra Firdani, Hee-Jung Chung, Mohy Uddin, Mina Hur, Jae Hyeon Park, Hyung Woo Kim, Anton Gradišek, Erik Dovgan

**Affiliations:** 1 Graduate Institute of Biomedical Informatics, Taipei Medical University, Taipei, Taiwan; 2 Department of Laboratory Medicine, Konkuk University School of Medicine, Seoul, South Korea; 3 Executive Office, King Abdullah International Medical Research Center, King Saud Bin Abdulaziz University for Health Sciences, Ministry of National Guard - Health Affairs, Riyadh, Kingdom of Saudi Arabia; 4 Department of Laboratory Medicine, Seoul National University Hospital, Seoul, South Korea; 5 Department of Internal Medicine, College of Medicine, Institute of Kidney Disease Research, Yonsei University, Seoul, South Korea; 6 Department of Intelligent Systems, Jožef Stefan Institute, Jamova cesta 39, 1000 Ljubljana, Slovenia; 7 CPBMI Consortium, Biomedical Informatics Training and Education Center of Seoul, National University Hospital, Seoul, South Korea

Correction to: *Scientific Reports* 10.1038/s41598-020-61247-0, published online 16 March 2020

This Article contains a typographical error in the Methods section where,

“We performed the hematologic analysis using Mindray BC-6800 (Mindray, Shenzhen, China) automated hematology analyzer that yielded CPD including CBC, leukocyte differentiation and reticulocyte count with information on volume, conductivity and different scatter measures^23^.”

should read:

“We performed the hematologic analysis using Mindray BC-6800 plus (Mindray, Shenzhen, China) automated hematology analyzer that yielded CPD including CBC, leukocyte differentiation and reticulocyte count with information on volume, conductivity and different scatter measures^23^.”

Also, the Article contains an error in Table 6 where the row entitled ‘Ramdom Forest (RF)’.

should read:

‘Random Forest (RF)’.

